# Clinical translation of anti-inflammatory effects of *Prevotella histicola* in Th1, Th2, and Th17 inflammation

**DOI:** 10.3389/fmed.2023.1070433

**Published:** 2023-05-05

**Authors:** Andrea Itano, Douglas Maslin, Kritika Ramani, Golbarg Mehraei, Nancy Carpenter, Taylor Cormack, Mahdi Saghari, Matthijs Moerland, Erin Troy, Will Caffry, Leslie Wardwell-Scott, Stuart Abel, Duncan McHale, Mark Bodmer

**Affiliations:** ^1^Evelo Biosciences, Cambridge, MA, United States; ^2^Centre for Human Drug Research (CHDR), Leiden, Netherlands

**Keywords:** small intestine, psoriasis, atopic dermatitis, Th1, Th2, Th17, inflammation, KLH

## Abstract

**Introduction:**

EDP1815 is a non-colonizing pharmaceutical preparation of a single stain of *Prevotella histicola* isolated from the duodenum of a human donor. We report here preclinical and clinical studies showing that the action of EDP1815, an orally delivered and gut restricted single strain of commensal bacteria can regulate inflammatory responses throughout the body.

**Methods:**

Supported by evidence for anti-inflammatory activity in three preclinical mouse models of Th1-, TH2-, and Th17-mediated inflammation, EDP1815 was tested clinically in three Phase 1b studies in patients with psoriasis, patients with atopic dermatitis, and healthy volunteers in a KLH skin challenge model.

**Results:**

Preclinically, EDP1815 was efficacious in all three mouse models of inflammation, showing reduction in skin inflammation as well as related tissue cytokines. In the Phase 1b studies, EDP1815 was found to be well tolerated by participants, with a safety profile comparable to placebo, including no severe or consistent side-effects reported, and no evidence of immunosuppression with no opportunistic infection occurring in these studies. In psoriasis patients, signs of clinical efficacy were seen after 4 weeks of treatment, which continued beyond the treatment period in the higher-dose cohort. In atopic dermatitis patients, improvements were seen throughout the key physician-and patient-reported outcomes. In a healthy-volunteer study of a KLH-induced skin inflammatory response, consistent anti-inflammatory effects were seen in two cohorts through imaging-based measures of skin inflammation.

**Discussion:**

This is the first report demonstrating clinical effects from targeting peripheral inflammation with a non-colonizing gut-restricted single strain of commensal bacteria, providing proof of concept for a new class of medicines. These clinical effects occur without systemic exposure of EDP1815 or modification of the resident gut microbiota, and with placebo-like safety and tolerability. The breadth of these clinical effects of EDP1815, combined with its excellent safety and tolerability profile and oral administration, suggests the potential for a new type of effective, safe, oral, and accessible anti-inflammatory medicine to treat the wide range of diseases driven by inflammation.

**Clinical Trial Registration**: EudraCT # 2018-002807-32; EudraCT # 2018-002807-32; NL8676; https://clinicaltrials.gov/ct2/show/NCT03733353; http://www.trialregister.nl.

## Introduction

The small intestine (SI) is an immunological window on the environment. Its mucosal surfaces must tolerate required foreign antigens that are absorbed as nutrients while protecting against toxic antigens and pathogens. Immune cells are found throughout the SI epithelial lining, both in specialized tertiary immune structures called Peyer’s patches and within the lamina propria and associated mesenteric lymph nodes (1). These mucosal surfaces are also colonized by a low density of commensal microorganisms which are distinct from the bulk of the colonic microbiota in their abundance, microenvironment, and taxonomic range (2, 3). Individual strains of microbes sampled from the mucosal surface of the small intestine have been shown to alter the phenotype of antigen presenting and immune effector cells in human *in vitro* cell experiments and to have anti-inflammatory effects in murine *in vivo* models of inflammation (4). The *in vivo* effects are not dependent on mucosal colonization; indeed, non-viable bacteria can induce these systemic effects, suggesting that signals generated by intestinal cells upon recognition of structural features on the surface of the microbes initiate the anti-inflammatory effect (4). Bacterial surface structures, such as capsular Polysaccharide A from *Bacteroides fragilis* have been previously described and shown to modulate local immune cell responses as well as systemic inflammatory responses (5, 6). The ability to modify systemic inflammation without systemic exposure confirms a link between mucosal and systemic immunology. Harnessing this link through pharmacological modulation offers the potential to create a new class of therapeutics which can modify systemic immunology without the need and risk of systemic exposure.

EDP1815 is prepared from a single strain of *Prevotella histicola* (*P. histicola*), which is a gram-negative, non-sporulating, obligate commensal anaerobe isolated from a duodenal biopsy of a human donor. *Prevotella* species have been found in the oral, nasopharyngeal, gastrointestinal, and genitourinary mucosal surfaces of all human populations tested to date (7). Abundance in stool can range from <1% to nearly 50% of total fecal microbial load (8).

EDP1815 drug product is manufactured from a master cell bank by fermentation and subsequent lyophilization and encapsulation. The drug substance is a lyophilized powder which is rendered essentially non-viable and non-colonizing during the manufacturing process after fermentation, with a cell viability of <0.02%. It has not been genetically modified.

This specific *P. histicola* strain was selected for its pharmacological anti-inflammatory properties using *in vitro* and *in vivo* models in a similar way to the discovery of conventional small molecule or biologic drugs. Preclinical studies in models of rheumatoid arthritis (CIA) (9) and experimental acute encephalomyelitis (EAE) (10) have shown that oral treatment with this strain of *P. histicola* has immunomodulatory effects leading to reduced inflammation development and severity. Furthermore, its beneficial effects in murine models of celiac disease (11), and of type 1 diabetes (12) have also previously been published. Mechanistic studies in mice have been conducted to show that these effects occur without systemic exposure and are due to the connectivity between mucosal and systemic immune networks (4).

To investigate whether the immunomodulatory activity of a non-colonizing microbial therapeutic can drive pharmacological effects which translate from mice to humans, we tested orally administered EDP1815 for its ability to modulate inflammation in a range of preclinical and clinical studies. We describe broad and potent anti-inflammatory effects of EDP1815 in preclinical studies which capture Th1, Th2 and Th17 biology and in three corresponding clinical studies that determined the safety and efficacy of EDP1815 in, (1) a T cell-mediated skin challenge model in healthy volunteers; (2) patients with atopic dermatitis, predominantly Th2-driven; and (3) patients with psoriasis, predominantly Th17-driven, demonstrating translation from mice to humans for a broadly acting, oral, immunomodulatory, non-colonizing microbial strain targeting the small intestine.

The delayed-type hypersensitivity (DTH) reaction, also known as type IV hypersensitivity reaction, is a common model of T cell-mediated inflammation in mice and other mammals. It is used for evaluating cell-mediated immune responses associated with CD4^+^ or CD8^+^ T cell reactivity, studying the mechanisms of skin inflammation, and evaluating therapeutic efficacy. Multiple effector mechanisms are involved but it is generally considered to be predominantly driven by Th1 cells (13) with some Th2 cell involvement (14). A similar T cell-mediated response can also be induced in humans using a neo-antigen skin challenge, with a resulting delayed-type hypersensitivity response quantified by an increase in skin blood perfusion and erythema (15, 16). Therapeutic interventions that target T cells inhibit both the mouse and human KLH delayed-type hypersensitivity response by a similar mechanism, and therefore the mouse model can be used to predict T cell-mediated responses in humans (17, 18).

Atopic dermatitis (AD) has a prevalence of 11–30% in children (19, 20) and 2–10% in adults (20) with the majority of patients having mild to moderate disease. Genetic predisposition, disruption of the epidermal barrier and immune dysregulation are components in the development of AD (21). Barrier disruption leads to skin inflammation and allergic sensitization driven by activation of T-cell subsets, predominantly Th2 immune responses. Th2 cytokines IL-4 and IL-13 drive chemokine production, further epidermal barrier dysfunction and allergic inflammation (22). Clinical data from studies with monoclonal antibodies including Dupilumab and Lebrikizumab have validated the role of anti-interleukin (IL)-4 and anti-IL-13 therapy in moderate to severe atopic dermatitis (23) though these therapies are limited to use in patients with moderate to severe disease due to challenges related to safety, convenience, and high cost. Another Th2 cytokine, IL-31, has been reported to increase production of cytokines and chemokines from skin cells, thereby inducing itch and pruritic skin lesions (24, 25). EDP1815 was tested in patients with atopic dermatitis to confirm its potential in treating Th2 diseases such as atopic dermatitis, allergy, and asthma.

Psoriasis is a chronic immune-mediated inflammatory disease with predominant pathological effects in the skin and musculoskeletal tissue with an adult prevalence of up to 2% (26). Similar to atopic dermatitis, most patients with psoriasis suffer from mild to moderate disease. It is characterized by psoriatic plaques and acanthosis due to uncontrolled keratinocyte proliferation. Disruptive cutaneous immune responses are responsible for the sustained inflammation seen in the psoriatic skin (27). As well as cutaneous features, it is associated with nail disease, arthritis, and metabolic syndrome (25). Infiltration of inflammatory dendritic cells drives the initial stages of disease followed by activation of Th17 cells. Th17, Th2, and Th1 cells have been noted in psoriatic lesions. The immuno-pathophysiology associated with psoriasis involves overexpression of IFN, TNF, IL-17, IL-20, and IL-22 (28). Clinical data with therapeutic monoclonal antibodies have validated the role of anti-TNF, antiIL-17 and anti-IL-23 therapy in moderate to severe psoriasis (27).

Here we describe for each of the Th1, Th2, and Th17 inflammation subtypes the translation from mice to an equivalent human model or disease with the aim of providing proof of concept for a novel treatment approach to resolve systemic inflammation through the small intestinal axis.

## Results

### EDP1815 is effective in a pre-clinical model of Th1-predominant inflammation

**Figure 1:**
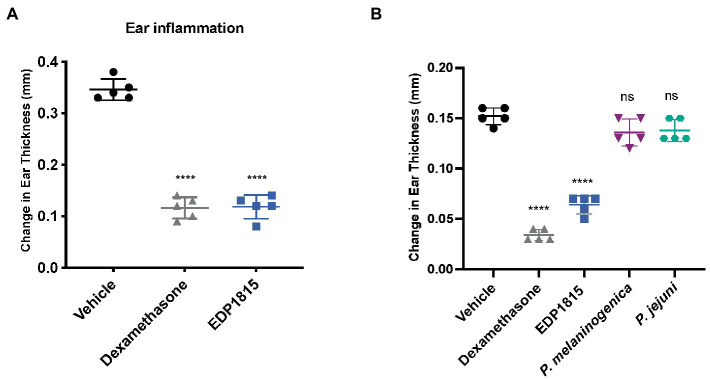
EDP1815 resolves Th1-driven inflammation *in vivo*. **(A)** In a DTH model, C57BL/6 mice were immunized with KLH and CFA on day 0 and challenged in the ear 4 weeks later with KLH. Mice were orally dosed for 5 days per week from the day after immunization through ear challenge with vehicle or EDP1815 (TCC-4.69E+09) or with dexamethasone systemically. Ear inflammation was measured 24 h post ear challenge. Data shown as change in ear thickness (*n* = 5 mice/group). **(B)** DTH model was set up as previously described. Mice were challenged in the ear 9 days after sensitization. Ear inflammation was measured 24 h post ear challenge. Data shown as change in ear thickness (*n* = 5 mice/group) for groups dosed with EDP1815 and other *Prevotella* strains (*P. jejuni* TCC-6.29E+09, *P. melaninogenica* TCC-2.48E+09). All experiments were performed twice. Data shown are representative and results are expressed as mean ± SEM. ****p < 0.0001, ns: not significant as determined by ordinary One-Way ANOVA.

To determine the therapeutic potential of orally delivered EDP1815 in Th1-driven inflammation a murine DTH was performed. Mice were sensitized subcutaneously with keyhole limpet hemocyanin (KLH) on the back and subsequently dosed with EDP1815 by oral gavage for 4 weeks and given an ear challenge with KLH on the day 29. 24hr later, ear swelling was measured as a marker of inflammation. Treatment with EDP1815 significantly reduced ear swelling compared with the vehicle-treated group (Figure 1A). Furthermore, in a shorter model, mice were dosed with EDP1815 by oral gavage for 8 days, given a KLH challenge on the ear on the ninth day and ear swelling was measured 24 h post challenge. EDP1815 was the most efficacious strain in lowering ear inflammation compared to strains of other closely related *Prevotella* species (Figure 1B).

### EDP1815 is effective and well-tolerated in a clinical model of Th1-predominant inflammation

EDP1815-102 was a phase 1b, single-center, randomized placebo-controlled study investigating the potential of EDP1815 to modulate Th1-driven inflammation in healthy human volunteers. Participants were dosed with EDP1815 for 28 days. Immunological sensitization to KLH was induced by intramuscular injection on day 3 of dosing followed by intradermal KLH challenge on day 26. Inflammation was assessed using specialized imaging techniques to measure antigen specific responses to KLH challenge comparing drug-treated and placebo participants, expressed in arbitrary units (AU) of Laser Speckle Contrast Imaging (LSCI) of basal flow and flare. Skin color and average redness was assessed by multispectral imaging. These quantitative endpoints were measured just before and then 2 days following the intradermal KLH challenge. Thirty two subjects were enrolled in 2 cohorts. In each cohort, 12 subjects received EDP1815, and 4 received matching placebo for 28 days. Active subjects in both cohorts were administered with 8.0 × 10^11^ cells of EDP1815, once daily as either 10 (cohort 1) or 5 capsules (cohort 2). Participants in cohort 2 were fasted 2 h pre-dose.

**Figure 2:**
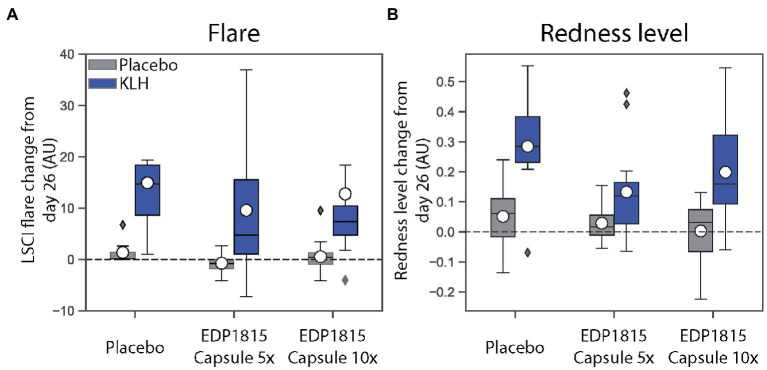
EDP1815 leads to reductions in inflammation measurements in a randomized double-blind trial of healthy volunteers administered KLH challenge. The effect of EDP1815 on the systemic immune system was evaluated in a double-blind, randomized, placebo-controlled trial using a KLH challenge. Two cohorts of 16 patients randomized 3:1 active (*n* = 12) to placebo (*n* = 4) were treated once daily for 28 days, with intramuscular administration of KLH on day 3, followed by intradermal KLH re-challenge on day 26. On the contralateral arm, placebo was administered intradermally on day 26 to account for local inflammation due to the injection. **(A)** Cutaneous microcirculation as a marker of inflammation was assessed by laser speckle contrast imaging to measure flare, expressed in arbitrary units (AU), at baseline and day 26. **(B)** Cutaneous erythema as a marker of inflammation was assessed by multispectral imaging to quantify redness, expressed in arbitrary units (AU), at baseline and day 26.

Although the study was not powered to detect statistically significant differences across treatment groups, notable trends corresponding to a reduction in inflammation as measured by dermal imaging were observed in the groups treated with EDP1815 in comparison to placebo. This was observed for all measurements: LSCI basal flow and flare (Figure 2A), and multispectral imaging skin color and redness (Figure 2B). The effects were consistent across measures and reproduced in the two cohorts. Given subject numbers the effect size did not reach statistical significance.

EDP1815 was safe and well tolerated with no overall difference in adverse events from placebo. There were no severe adverse events in any participants (Table 1).

**Table 1:** EDP1815 combined safety data summary in healthy volunteers, psoriasis, and atopic dermatitis.

Adverse event	Skin challenge healthy volunteers			Psoriasis			Atopic dermatitis	
	Placebo (*n* = 8)	Active Cohort 1 (*n* = 12)	Active Cohort 2 (*n* = 12)	Placebo (*n* = 10)	Active low dose (*n* = 8)	Active high dose (*n* = 12)	Placebo (*n* = 8)	Active (*n* = 16)
Any TEAE								
Mild	8 (100%)	11 (92%)	8 (67%)	5 (50%)	4 (50%)	9 (75%)	6 (75%)	14 (88%)
Moderate	0	1 (8%)	0	5 (50%)	1 (13%)	3 (25%)	3 (13%)	3 (19%)
Severe	0	0	0	0	0	0	0	0
Serious	0	0	0	0	0	0	0	0
Related TEAE								
Mild	4 (50%)	7 (58%)	2 (17%)	1 (10%)	3 (38%)	2 (17%)	1 (13%)	5 (31%)
Moderate	0	0	0	2 (20%)	0	1 (13%)	0	0
Severe	0	0	0	0	0	0	0	0
Serious	0	0	0	0	0	0	0	0
Death	0	0	0	0	0	0	0	0
Adverse Event reported by ≥ 3 patients in either group								
Headache	2 (25%)	7 (58%)	6 (50%)	3 (30%)	1 (13%)	2 (17%)	2 (25%)	8 (50%)
Fatigue	3 (38%)	1 (8%)	0	0	0	0	0	0
Myalgia	2 (25%)	4 (33%)	2 (17%)	0	0	0	0	0
Viral Upper respiratory tract infection	3 (38%)	3 (25%)	1 (8%)	0	0	0	0	0
Rash	0	3 (25%)	0	0	0	0	0	0
Abdominal pain upper	0	3 (25%)	0	0	0	1 (8%)	0	0
Abdominal pain	1 (13%)	0	0	0	0	0	0	3 (19%)
Diarrhea	0	0	0	0	1 (13%)	0	0	6 (38%)
Treatment Modification								
Discontinuation	–	–	–	0	0	0	0	0
Dose Interruption	–	–	–	0	0	0	0	1 (6%)
Dose Reduction	–	–	–	0	0	0	0	0

The preferred term was used to summarize the adverse events across the 3 groups. Different dictionaries may have been used between the healthy volunteer and psoriasis and atopic dermatitis study.

### EDP1815 is effective in a pre-clinical model of Th2-predominant inflammation

**Figure 3:**
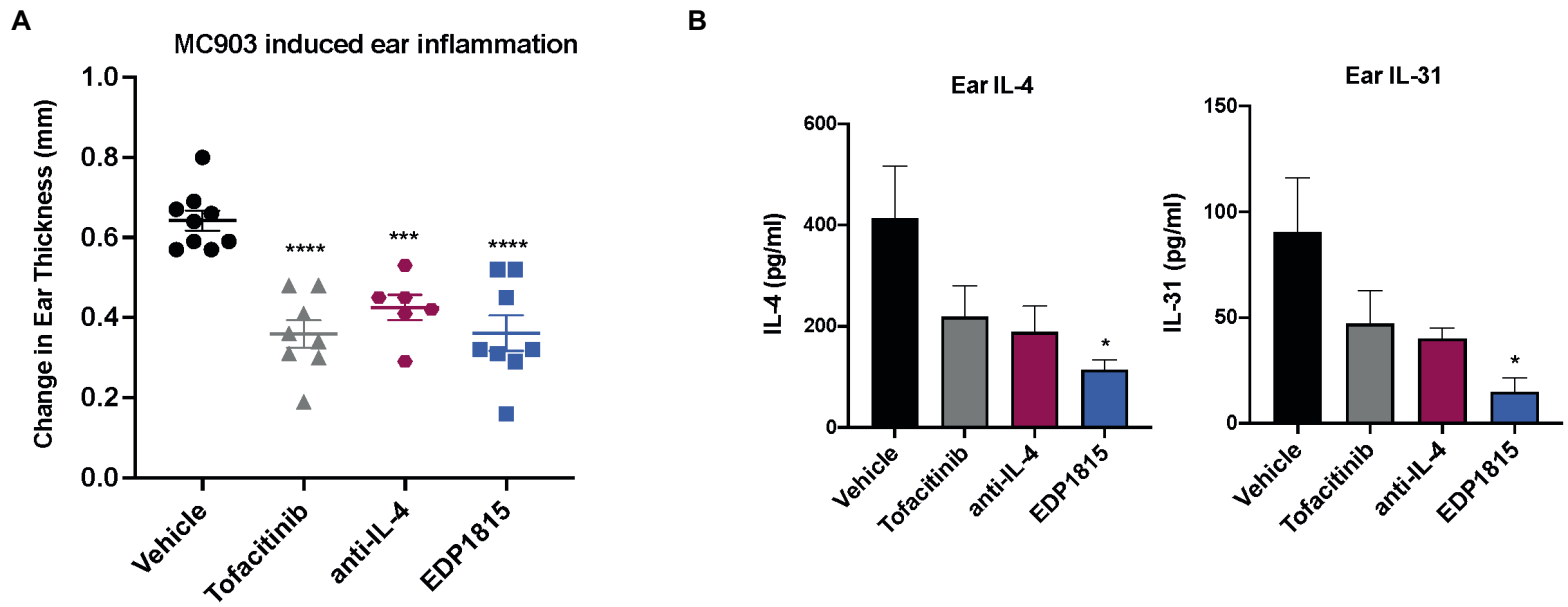
EDP1815 is protective in Th2 driven model of allergic inflammation. BALB/c mice were topically sensitized daily on the ear with 45 nM MC903 from day 1 to 14. Mice were dosed orally daily with vehicle or EDP1815 (TCC-3.13E+09). Ear inflammation was measured on day 14. **(A)** Change in ear thickness (*n* = 5 mice/group). **(B)** Upon termination of study, ears were homogenized, and protein levels of IL-4 and IL-31 were measured by MSD. All experiments were performed twice. Data shown are representative and results are expressed as mean ± SEM. *p < 0.05, ***p < 0.001, ****p < 0.0001 as determined by Ordinary one-way ANOVA.

The vitamin D3 analog, MC903, can be used in mice to generate Th2-driven epidermal inflammation with increased dermal cell infiltrates consisting of eosinophils, T cells, neutrophils, and mast cells. Following application of MC903, skin shows increased levels of a range of Th2 cytokines including IL-4, IL-5, IL-13, and IL-31 (29). BALB/c mice were sensitized on the ears with MC903 for 14 days and dosed orally daily with EDP1815 for 14 days. Treatment with EDP1815 resulted in significantly lower ear inflammation in comparison with vehicle-treated animals, on par with tofacitinib, an oral JAK-1/3 inhibitor, and a systemic antibody blocking IL-4 (Figure 3A). In animals treated with EDP1815, *ex vivo* analysis revealed a reduction in levels of IL-4, a central Th2 cytokine, as well as of IL-31 (Figure 3B).

### EDP1815 is effective and well-tolerated in atopic dermatitis, a condition with Th2-predominant inflammation

To test the potential of EDP1815 to treat Th2-driven inflammatory disease, it was evaluated in a phase 1b clinical study (EudraCT # 2018-002807-32). A cohort of 24 participants with mild and moderate atopic dermatitis was randomized 2:1 active: placebo. 8.0 × 10^11^ total cells of EDP1815 was administered once daily for 56 days, with a follow-up visit after 14 days off drug on Day 70. The primary endpoint was safety and tolerability of EDP1815. Secondary endpoints included physician-rated scales of atopic dermatitis severity (EASI, IGA, BSA, IGA*BSA, and SCORAD); as well as patient-reported outcomes (Pruritus-NRS, DLQI, and POEM). Baseline mean EASI and IGA scores were 8.31 and 2.63 respectively, for the 16 patients receiving EDP1815, and 9.31 and 2.7, respectively for the 8 patients receiving placebo.

EDP1815 had a placebo like safety and tolerability profile with no treatment-related adverse events of moderate or severe intensity, and no serious adverse events (Table 1).

**Figure 4:**
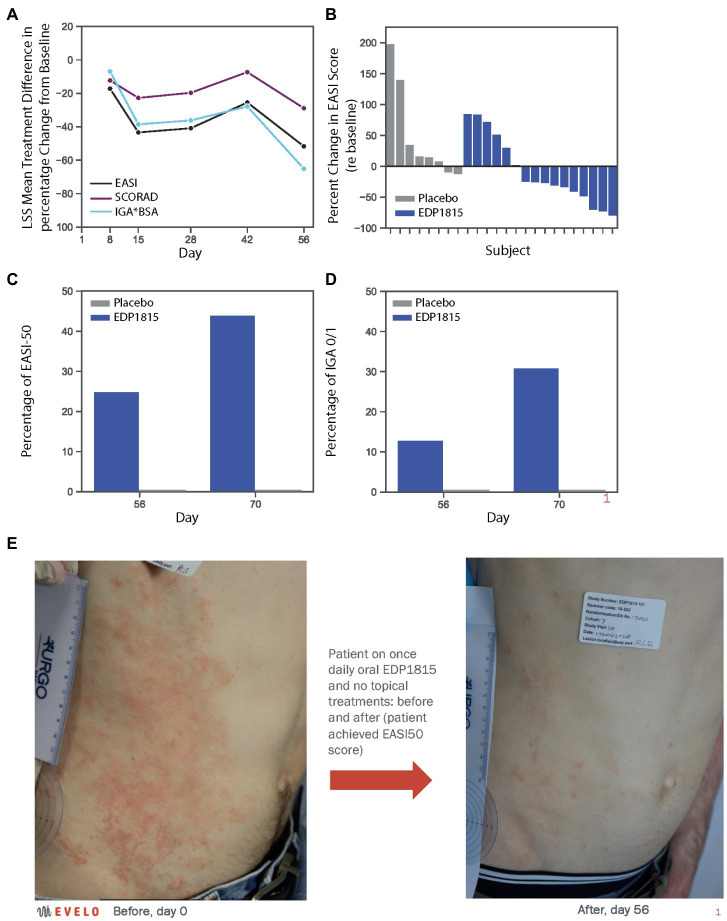
EDP1815 leads to clinical improvements in a randomized double-blind trial of patients with atopic dermatitis. A phase 1b cohort of 24 patients with mild and moderate atopic dermatitis were randomized to EDP1815 (*n* = 16) or placebo (*n* = 8) and treated once daily for 56 days, with follow-up off treatment at day 70. **(A)** Clinical parameters of atopic dermatitis were measured at baseline and treatment days 8, 15, 28, 42, and 56. The treatment difference was calculated by subtracting the mean percentage change from baseline in placebo patients from that in active patients at each time point, and for each of the key clinical scores quantifying disease severity: EASI, IGA*BSA, and SCORAD. At day 56, the treatment difference for EASI was 52% (*p* = 0.062), for IGA*BSA was 65% (*p* = 0.022), and for SCORAD was 35% (*p* = 0.068). **(B)** Waterfall plot, with each participant’s percentage change from baseline in the EASI score at day 56 represented by each bar. Two placebo patients saw improvement, compared to ten patients randomized to EDP1815, with 4 patients achieving EASI50 or better at this timepoint. **(C)** Proportion of patients achieving EASI50 threshold or better, in active versus placebo group patients at day 56 (25% vs. 0%, respectively) and day 70 (44% vs. 0%, respectively). **(D)** Proportion of patients achieving IGA0/1 threshold in active versus placebo group patients at day 56 (13% vs. 0%, respectively) and day 70 (31% vs. 0%, respectively). **(E)** Photographs taken of a subject receiving EDP1815 and no topical or other active atopic dermatitis treatment in this study, at baseline and after 56 days of treatment. Significant improvements in erythema, papulation and excoriations are visible. The patient achieved an EASI improvement of 50%, from 9.8 at baseline to 4.9 at day 56.

The differences in percentage decrease from baseline in EASI, IGA*BSA and SCORAD between the EDP1815 treated group and the placebo group were 52% (*p* = 0.062), 65% (*p* = 0.022), and 35% (*p* = 0.068), respectively (Figure 4A). 10 of 16 patients receiving EDP1815 saw improvements in their EASI score at day 56, compared to only 2 out of 8 patients receiving placebo (Figure 4B).

At the day 70 follow-up visit, further clinical improvements were observed in the EDP1815 treated group. The percentage of patients receiving EDP1815 achieving EASI50 was 44% compared with 0% in the placebo group (Figure 4C); and the proportion achieving an IGA score of 0 or 1 was 31%, with 0% again in the placebo group (Figure 4D). Figure 4E shows a representative clinical improvement of skin condition in a participant receiving EDP1815, and no other oral or topical treatments, for 56 days in this trial. This was an EASI50 response, with an improvement from an EASI score of 9.8 at baseline to 4.9 at day 56. In addition to the clinical improvements in the physician rating scales, this participant’s patient reported outcomes (PROs) also improved with the DLQI score moving from 13 (severe impact) to 1 (no impact), and the POEM score from 22 (self-rating of ‘severe eczema’) to 5 (‘mild eczema’) at day 56.

The mean individual PRO improvement from baseline in the DLQI (−3.6) and POEM (−4.1) in EDP1815-treated patients at day 56 exceeded the minimally clinically important difference thresholds (30, 31) and exceeded the placebo group changes (−0.3 and + 1.6, respectively). Mean improvements in itch and sleep were seen within all scales measuring these parameters (Pruritis-NRS, DLQI, SCORAD, and POEM) at the end of the treatment period.

These results provide proof of concept that EDP1815 can resolve Th2-driven inflammation with a placebo-like safety and tolerability profile in patients with atopic dermatitis.

### EDP1815 is effective in a pre-clinical model of Th17-predominant inflammation

**Figure 5:**
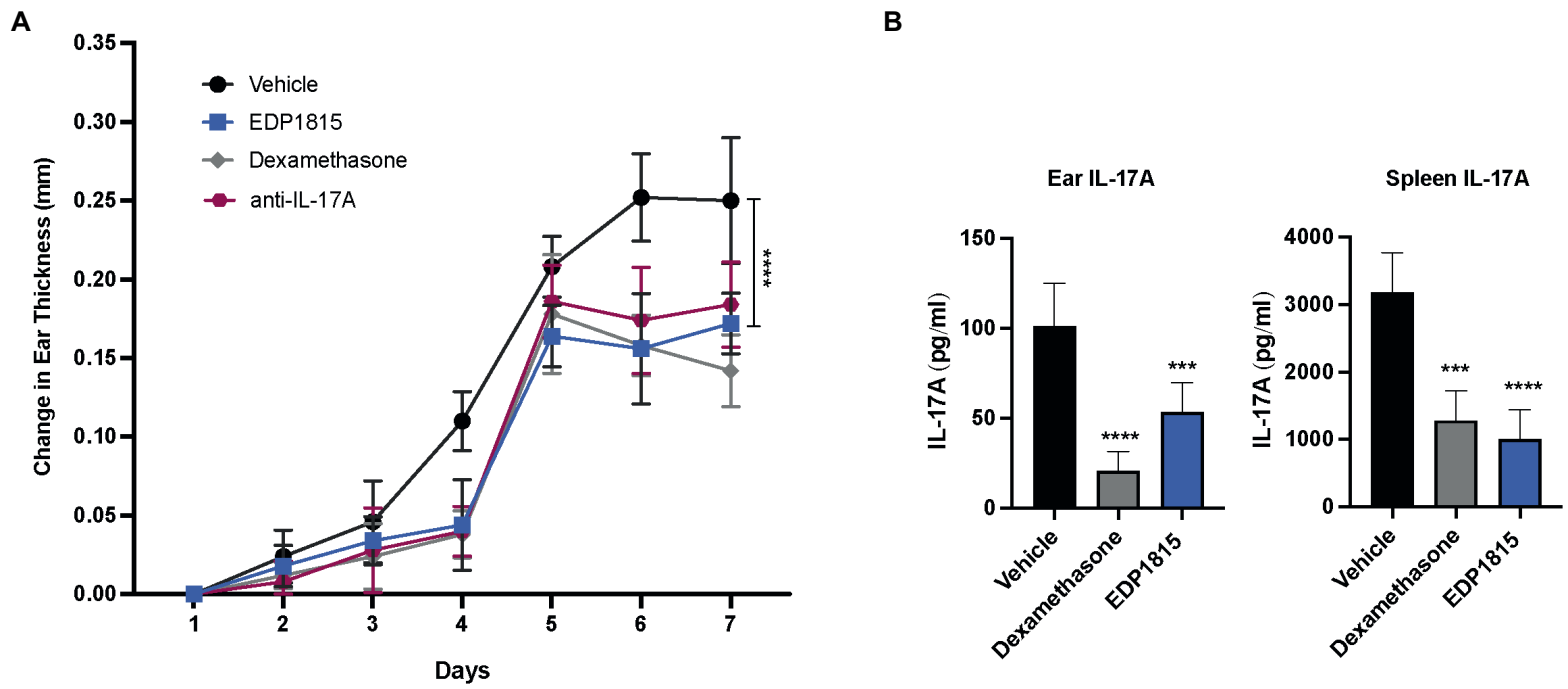
EDP1815 alleviates skin pathology Th17 model of cutaneous inflammation. BALB/c mice were topically treated with 5% imiquimod, a TLR7 agonist for 7 days on the back skin and ear. Mice were dosed orally daily from day 1 through 7 with vehicle, dexamethasone (1 mg/kg IP) or EDP1815 (TCC-3.13E+09). **(A)** Time course of change in ear inflammation over 7 days. **(B)** At termination, splenocytes were *ex vivo* re-stimulated with PMA/Ionomycin for 48 h. Protein levels of IL-17A was measured from supernatants by MSD. IL-17A protein levels were also measured in ear tissue homogenates. All experiments were performed twice. Data shown are representative and results are expressed as mean ± SEM. ***p < 0.001, ****p < 0.0001 as determined by Ordinary one-way ANOVA.

A TLR7 agonist, imiquimod, induces clinical and histological changes characteristic of human psoriasis, including epidermal thickening, scaling and erythema (32). Mice were sensitized on the ear and back with imiquimod cream daily for 7 consecutive days and dosed daily with oral EDP1815, vehicle, dexamethasone, or anti-IL17A antibody. Ear thickness was a measure of inflammation. Treatment effects in animals dosed with EDP1815 were seen as early as 4 days after the start of IMQ application and were comparable to those observed in animals treated with dexamethasone or anti-IL-17A in reducing ear thickness as well as back inflammation (Figure 5A). At termination on day 8, IL-17A protein levels in the ear tissue were reduced by treatment with EDP1815 in comparison to vehicle (Figure 5B). IMQ is known to also induce an increase in IL-17A production in splenocytes (32). *Ex vivo* re-stimulation of splenocytes with PMA/Ionomycin showed decreased production of IL-17A in mice treated with EDP1815 (Figure 5B).

### EDP1815 is effective and well-tolerated in psoriasis, a clinical disease of Th17-inflammation

To determine the potential of EDP1815 to treat Th17-driven inflammatory disease it was evaluated in two parallel cohorts of a phase 1b clinical study in patients with psoriasis (EudraCT # 2018-002807-32). Adult patients with mild to moderate chronic plaque psoriasis were randomized 2:1 to receive EDP1815 or matching placebo capsules. Doses were 1.6 × 10^11^ (cohort 3) and 8.0 × 10^11^ (cohort 4) bacterial cells, once daily for 28 days, with follow-up after 14 days off treatment at day 42.12 patients were dosed with the lower dose, and 18 with the higher dose. Placebo subjects were pooled across both cohorts. The primary endpoint was safety and tolerability of EDP1815. Secondary endpoints included physician-rated scales of psoriasis: Psoriasis Area and Severity Index (PASI) and Lesion Severity Score (LSS). Baseline mean PASI scores were 9.5 (cohort A-3), 6.2 (cohort 4), and 6.7 (pooled placebo cohorts). Mean LSS scores at baseline were 8.1 (cohort 3), 7.8 (cohort 4), and 7.8 (pooled placebo cohorts).

The primary endpoint safety data showed EDP1815 to have a safety and tolerability profile comparable to placebo (Table 1). As for the other studies reported here, there were no serious adverse events, and no adverse events of severe intensity.

**Figure 6:**
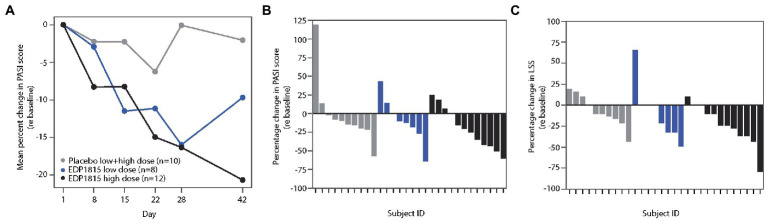
EDP1815 leads to clinical improvements in two randomized double-blind cohorts of patients with psoriasis. Two parallel phase 1b cohorts of 12 and 18 patients with mild and moderate psoriasis were randomized to EDP1815 or placebo in a 2:1 ratio, and treated once daily for 28 days, with follow-up off treatment at day 42. **(A)** Mean percentage change from baseline in PASI score from baseline to final follow-up visit. At the end of treatment visit on day 28, the mean percentage reduction in PASI for EDP1815 cohorts was 16%, compared to 0.1% for placebo. **(B)** Waterfall plot, with each participant’s percentage change from baseline in the PASI score at day 42 represented by each bar. EDP1815 low and high dose compared to pooled placebo. A 25% or greater improvement in PASI was observed in 1 of 10 placebo patients, 2 of 8 participants receiving low dose EDP1815, and 6 of 12 participants receiving high-dose EDP1815. **(C)** Waterfall plot, with each participant’s percentage change from baseline in the LSS score at day 42 represented by each bar. EDP1815 low and high dose compared to pooled placebo. A 25% or greater improvement in LSS was observed in 1 of 10 placebo patients, 3 of 8 participants receiving low dose EDP1815, and 7 of 12 participants receiving high-dose EDP1815.

The PASI score is a composite measure of psoriasis plaque severity, and body coverage (33). Following 28 days of treatment, the mean percentage reduction in PASI for EDP1815 cohorts was 16%, compared to 0.1% for placebo. At day 42, the percentage improvement from baseline in cohort 2 active participants increased further to 21% (Figure 6A). In this cohort, 6 of the 12 patients achieved a 25% improvement in PASI or better at day 42, compared to 1 of 10 in placebo (Figure 6B).

LSS measures the severity of a target plaque using scaling, erythema, and plaque elevation, giving a maximum total score of 12. The mean percentage reductions in LSS scores at day 28 were 23 and 15% in the low-and high-dose cohorts respectively, compared to a 1% increase from baseline in the placebo group. Figure 6C shows the individual percentage changes in LSS from baseline at day 42, with 7 of the 12 patients in the high-dose cohort achieving 25% improvement or greater, compared to 1 of 10 in the placebo group.

These two comparable sets of clinical data provide proof of concept that EDP1815 can drive clinical improvements and resolve inflammation in the skin of patients with psoriasis with a placebo-like safety and tolerability profile. As seen in atopic dermatitis, responses were continuing to improve and had not reached peak effect at the end of the dosing period.

### EDP1815 is gut-restricted with no systemic absorption and no impact on background microbiome

To determine the biodistribution of EDP1815 following oral dosing in mice, strain-specific primers were designed to differentiate EDP1815 from other *P. histicola* strains. This enabled sensitive tracking of EDP1815 in mouse experiments in the potential presence of alternate species of *P. histicola* in the background gastrointestinal microbiome.

Following oral administration of a single dose, EDP1815 was transiently detected in the GI tract and stool. EDP1815 was detected in the intestine and stool for up to 8 h and not at 16 h post-administration, showing that the lyophilized microbes did not colonize the gut. Importantly, EDP1815 was not detected outside of the GI tract at any time point. These data demonstrate that EDP1815 is luminally restricted with undetectable systemic exposure following oral dosing in mice (Supplementary Figure S1).

Evidence of systemic exposure of EDP1815 in humans was evaluated in the phase 1b clinical cohorts of psoriasis patients described previously. EDP1815 was not detected in blood by PCR or by culture at any time-point during the dosing period, through completion of the 28 days of dosing.

In these psoriasis cohorts EDP1815 was not detected by qPCR in stool samples taken 14 days after completion of dosing. This experiment was repeated in the healthy volunteer DTH study. Again fecal concentrations of EDP1815 were below the limit of quantification pre-dose and 5 days post last dose in all treatment groups, confirming a lack of gut colonization.

**Figure 7:**
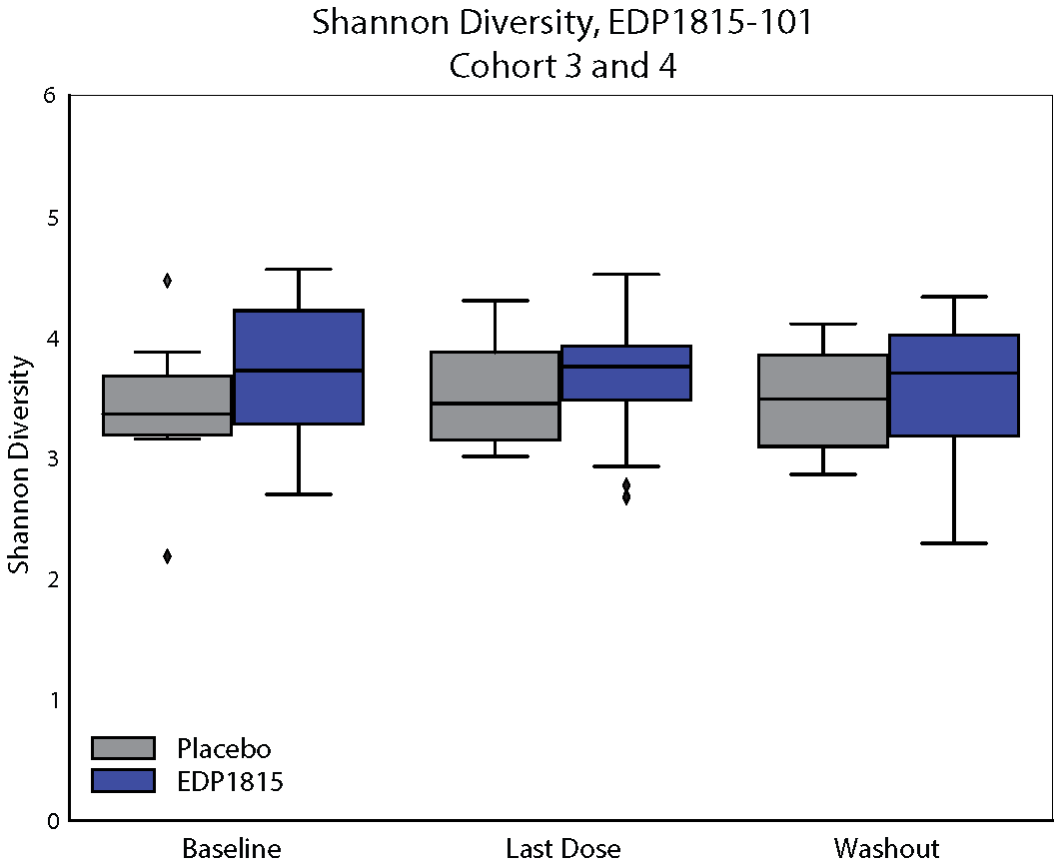
EDP1815 transits through the gastrointestinal tract between 8 and 16 h. 16S PCR analysis of stool samples taken from patients at baseline, day 28 and day 42. The top 10 genera by percentage composition are given for EDP1815 and placebo across the two cohorts at each timepoint. There are no significantly differentiated genera for EDP1815 treated individuals, or significant differences between EDP1815 and placebo.

Finally, 16S ribosomal RNA sequencing of stool samples confirmed that the pharmacodynamic activity observed with EDP1815 was not due to secondary alterations in the colonic microbiome. Samples taken at baseline, on drug and at follow up showed no significant changes in the Shannon Index (diversity) or composition from baseline either during or following cessation of dosing (Figure 7). Furthermore, no significant changes in fecal microbe abundance at the genera level were detected for either placebo or EDP1815 dosed subjects when comparing between time points (Supplementary Figure S2). Microbes from the *Bacteroides* and *Blautia* genera were the most abundant in the fecal microbiome for all groups, and for all groups the 10 most abundant genera made up more than 90% of the microbiome by percent composition.

These data confirm that the resolution of inflammation observed with EP1815 is not due to systemic exposure, gut-colonization, nor indirect effects on the colonic microbiome.

## Discussion

Here we describe the translational development of EDP1815, a single strain of *Prevotella histicola*, and demonstrate that it can modify systemic inflammation in humans across Th1, Th2 and Th17 driven inflammation through its action in the gut. The ability of gut-restricted EDP1815 in the mouse DTH study (Figure 1) to reduce a distal inflammatory response to the same degree as systemic-administered dexamethasone is remarkable. It demonstrates a link between the enteric and systemic immune systems which is effective at a level which has not been previously described and can be harnessed for pharmacological effects. It suggests an entirely novel approach to the treatment of a wide range of inflammatory diseases.

This is the first report of a single microbe that is able to pharmacologically modulate multiple distinct inflammatory pathways in preclinical models and in human studies. In a healthy-volunteer study of a KLH-induced skin inflammatory response, consistent anti-inflammatory effects were seen in two cohorts through imaging-based measures of skin inflammation. In psoriasis patients, signs of clinical efficacy were seen after 4 weeks of treatment, which continued beyond the treatment period in the higher-dose cohort. In atopic dermatitis patients, improvements were seen throughout the key physician-and patient-reported outcomes. In all these studies, EDP1815 was found to be well tolerated by participants, with a safety profile comparable to placebo, including no severe or consistent side-effects reported. Of note is the lack of any evidence of immunosuppression with no opportunistic infection occurring in these studies.

The mechanism by which EDP1815 impacts these pathways leading to inflammation resolution is under investigation. We recently published data using another orally delivered non-live microbial product, EDP18167, showing induction of peripheral T cells with an anti-inflammatory phenotype generated by the action of EDP1867 within the gut and the cellular trafficking mechanism that enables this (4). We have similar data for EDP1815 (manuscript in preparation).

Previously published studies have demonstrated that the strain of *Prevotella histicola* from which EDP1815 was developed can resolve both Th1-and Th17-mediated inflammation in mouse collagen-induced arthritis and autoimmune experimental encephalomyelitis (9, 10). Efficacy of oral treatment of mice with *P. histicola* was dependent on regulation by CD103+ dendritic cells and by generation of regulatory T cells in the gut, resulting in suppression of pro-inflammatory Th1 and Th17 responses and increased transcription of interleukin-10. And recently, oral administration of *P. histicola* was reported to delay the onset of type 1 diabetes in non-obese diabetic (NOD) mice (34). This effect correlated with a significant increase in regulatory T cells and decrease in NKp46+ cells in the pancreatic lymph nodes. These findings are in line with the preclinical studies described here, where treatment with EDP1815 led to a similar down-regulation of Th1 and Th17 cytokines, as well as Th2 cytokines in model of atopic skin inflammation. Commensal microbes have been shown to interact with and modulate cells of the gastrointestinal tract to influence local inflammation (35). These mechanisms include production of molecules such as bacterial metabolites or bioactive lipids and require colonization by the bacteria to exert their effects. However, data from the clinical studies described here show that the systemic effects of EDP1815 occur with no systemic absorption, no colonization, and no impact on the gut microbiome. Therefore, it is likely that direct interactions between EDP1815 and microbial pattern recognition receptor-expressing cells, such as intestinal epithelial cells and immune cells that can sample contents of the lumen, lead to the downstream systemic inflammation resolving effects described here. A regulatory mechanism that relies solely on direct interactions between the microbe and the cells of the intestine after oral administration would explain how EDP1815 can exert its effects with only transient occupancy of the gastrointestinal tract without systemic exposure.

One limitation of the study is that the psoriasis and atopic dermatitis cohorts comprised a relatively small number of patients. While the primary goal of these first in human studies was to establish safety and tolerability, the magnitude and consistency of the clinical effects of EDP1815 are encouraging. Another limitation is that the clinical studies were of relatively short duration. The mechanism of action proposed by the preclinical studies would predict that deeper responses would develop over time, as more regulatory T cells accumulate at sites of inflammation and continue to down-modulate effector Th1 and Th17 cells.

In conclusion, the preclinical activity of EDP1815 and the clinical proof of concept results demonstrate that EDP1815 has the potential to be an effective, safe, and well-tolerated oral anti-inflammatory therapy. The data presented here led to the further clinical development of EDP1815: in a phase 2 dose-ranging study in mild and moderate psoriasis [NCT04603027], and a phase 2 study of mild to severe atopic dermatitis [NCT05121480]. The data from the Phase 1b studies described here also suggest the potential of EDP1815 for the treatment of a wide range of inflammatory conditions, introducing a new class of medicines to the medical armamentarium.

## Materials and methods

### Mouse studies

#### Mice

Female BALB/c and C57BL/6 mice (6–8 weeks old) were purchased from Taconic Farms. Animals were housed in specific pathogen–free conditions in a vivarium (5 mice per cage), and all experiments were performed under Institutional Animal Care and Use Committee (IACUC) approved protocols and guidelines at Avastus Preclinical Services (Cambridge, MA). Mice were allowed to acclimate in the vivarium for 1–2 weeks prior to the start of experiments. Mice were monitored daily, provided PicoLab Rodent Diet 20 and autoclaved water *ad libitum*.

#### Dosing with EDP1815 and controls *in vivo*

For each *in vivo* study, EDP1815 aliquots were distributed into plastic test tubes with caps and stored at 4°C. Mice were treated orally with EDP1815 (specific TCC is noted in each figure legend) or vehicle control (anaerobic sucrose, PO) for duration of different models as described in figure legends. Dexamethasone (1 mg/kg, i.p., Sigma) was used as a positive control unless otherwise specified.

#### Delayed type hypersensitivity mouse model

Mice were immunized with 50 μL of emulsion of keyhole limpet hemocyanin (KLH) in Complete Freund’s Adjuvant (CFA) on four sites on the back. In a longer duration model, mice were dosed for 4 weeks and on day 29, mice were challenged with KLH (10 μg/10 μL) intradermally in the ear. In a shorter duration model, mice were dosed for 8 days and on day 9, mice were challenged with KLH (10 μg/10 μL) intradermally in the ear. Ear measurements were recorded 24 h post ear challenge using digital calipers. Change in ear thickness was expressed as ear thickness at 24 h post challenge minus ear thickness at baseline.

#### Imiquimod-induced psoriasis-like skin inflammation protocol

Mice were sensitized topically with 20 mg imiquimod cream (Aldara; 3M Pharmaceuticals, St Paul, MN, USA) on ears daily for 7 consecutive days. Ear measurements were taken daily using digital calipers and scores were reported as change in ear thickness calculated as ear score on day 8 minus baseline ear score on day 1.

#### MC903 driven atopic dermatitis

Mice were sensitized daily for 14 consecutive days with 45 nmol of MC903 (calcipotriol; Tocris Bioscience) in 20 μL of 100% EtOH on ears. Baseline ear measurements were taken prior to the first ear sensitization on day 1 using Digital Calipers (Fowler). On day 14, ear thickness was measured. Delta change in ear thickness was expressed as ear thickness at day 14 minus ear thickness at baseline.

#### Mouse *ex vivo* re-stimulation assays

Spleens were harvested at terminal time points and collected into 0.5 mL of cold, complete-RPMI (10% FBS, 1x Glutamax, 1 mM sodium pyruvate, 100 mM HEPES, 1x non-essential amino acids, 1x beta-mercaptoethanol, 1x antibiotic-antimycotic) (all reagents from Gibco). Single cell suspensions were prepared and 200,000 cells/well were plated. Cells were stimulated *ex vivo* with either LPS (200 ng/ml, Invivogen) or Poly I:C (Invivogen) for 48 h at 37°C and 5% CO2. Supernatants were collected at the end of stimulations and used for multiplex ELISAs of cytokine levels using Meso Scale Discovery kits. Ear tissues were dissociated in 250 μL T-PER buffer (Thermo Scientific) containing Halt Protease (Thermo Scientific) and protein was quantified with BCA kit (Thermo Scientific). 100 μg of protein was used to measure cytokine levels using MSD kits.

### Clinical studies

#### EDP1815 production and formulation

EDP1815 drug substance is freeze-dried *P. histicola* bacterial cells. EDP1815 drug product is manufactured as enteric-coated hydroxypropyl methylcellulose (HPMC) hard capsules in two strengths, 80 billion (8.0 × 10^10^) and 160 billion (1.6 × 10^11^) total cells per capsule. The capsule formulations of EDP1815 consist of drug substance, mannitol, magnesium stearate and colloidal silicon dioxide. Both dose strengths are enteric coated to protect EDP1815 from stomach pH degradation and designed for release at pH ≥ 5.5.

Corresponding placebo capsule formulation is manufactured using microcrystalline cellulose and magnesium stearate.

### Healthy volunteer KLH study design

This was a phase 1, randomized, placebo-controlled, double-blind, multiple dose study in thirty-two healthy volunteers performed at the Center for Human Drug Research (CHDR), Leiden, The Netherlands. The Declaration of Helsinki was the principle for trial execution. The independent Medical Ethics Committee “Medisch Ethische Toetsingscommissie van de Stichting Beoordeling Ethiek Biomedisch Onderzoek” (Assen, the Netherlands) approved the study prior to any clinical study activity. All subjects provided written informed consent before participation. The trial was registered on trialregister.nl (NL8676).

#### Subjects

Main inclusion criteria were healthy light skinned (Fitzpatrick skin type I-III) participants, 18 to 60 years of age with a body mass index between 18 and 35 kg/m^2^, and no known previous exposure to KLH. Health status was verified by recording a detailed medical history, a complete physical examination, vital signs, a 12-lead electrocardiogram (ECG) and laboratory testing (including hepatic and renal panels, complete blood count, fecal calprotectin, virology, and urinalysis). Subjects were excluded in case of any disease associated with immune or GI system impairment or use of prescription medication within 4 weeks prior to first dose.

#### Dose selection and regimen

The dose, 8.0 × 10^11^ total cells once daily, was based on the results of study EDP1815-101.

#### Study design and treatments

Subjects were enrolled into two cohorts. In each cohort, subjects were randomized to either EDP1815 or placebo (12:4). The first cohort received EDP1815 powder in enteric-coated capsules, supplied as 8.0 × 10^10^ total cell count per capsule, administered orally at a dose of 10 capsules daily for 28 days. The second group also received EDP1815 powder in enteric-coated capsules, however this was supplied as 1.6 × 10^11^ total cell count per capsule, administered orally at a dose of 5 capsules daily for 28 days. Intramuscular KLH immunization was performed in the deltoid muscle after three subsequent doses of study drug. KLH was administered in a formulation of 0.1 mg of subunit KLH (Immucothel^®^) adsorbed in 0.9 mg aluminium hydroxide (Alhydrogel^®^) into 0.5 mL NaCl 0.9%. Twenty-one days after intramuscular KLH administration, all subjects received an intradermal KLH administration in the left ventral forearm and placebo administration in the right ventral forearm. The formulation of 0.001 mg subunit KLH in 0.1 ml NaCl 0.9% used for intradermal administration and interval of twenty-one days between intramuscular KLH immunization and intradermal KLH administration and the interval of 48 h between baseline and follow up skin challenge response assessment was based on previous other studies (15, 36–40). Prior to, and 2 days after the intradermal KLH administration, the skin hypersensitivity response was quantified.

#### Safety and tolerability

Safety and tolerability were monitored by physical examination, assessment of vital signs, laboratory parameters (i.e., full blood count, biochemistry, serology, immunophenotyping, fecal calprotectin, and urinalysis) and ECG data from 12-lead ECGs at regular intervals. Subjects were monitored continuously for AEs.

#### Study treatment compliance

Compliance was assured by supervised administration of the study treatment during the in-clinic period. Administration at home was recorded by an electronic diary by means of photography of the capsules taken and recording the date and time.

#### Skin challenge response cutaneous blood perfusion

Cutaneous blood perfusion quantification was performed with laser speckle contrast imaging (LSCI; PeriCam PSI System, Perimed AB, Järfälla, Sweden) as previously described (15). In short, assessments were performed in a temperature-controlled room (22°C) after acclimatization of the subjects. LSCI recordings of the target area on the left and right ventral forearms were captured with the use of dedicated software (PimSoft, Perimed AB, Järfälla, Sweden). Circular regions of interest at the intradermal injection sites were defined and cutaneous blood perfusion (indicated as basal flow) was quantitatively assessed and expressed in arbitrary units (AUs). The homogeneity of cutaneous blood perfusion in the region of interest (indicated as flare), expressed as values that are +1 standard deviation (SD) from the mean basal flow within the region, was also quantitatively assessed and expressed in AUs.

#### KLH skin challenge erythema

Erythema quantification was performed with multispectral imaging (Antera 3D^®^, Miravex, Dublin, Ireland) as previously described (15). In short, the camera was placed on the target area on the ventral forearms and images were captured using dedicated software (Antera 3D^®^ software, Miravex, Dublin, Ireland). Circular regions of interest at the intradermal injection sites were defined and erythema was quantified using the average redness and CIELab a* Antera 3D^®^ software modalities expressed as AUs. The average redness modality displays the distribution of redness using an internal software algorithm and the CIELab a* value, which is part of the CIELab color space and expresses color as a numerical value on a green–red color scale.

#### Statistics

Subjects were randomized to EDP1815 or placebo in a 3:1 ratio. KLH skin challenge endpoints were analyzed with an analysis of covariance (ANCOVA) with treatment as fixed factor and the baseline and the change from baseline of the saline-injected control (right forearm) added as covariates. The general treatment effect and specific contrasts were reported with the mean change from baseline and SD. Fecal microbiome endpoints were analyzed using Python (Python Software Foundation, Wilmington, Delaware, US). The relative *Prevotella* abundance was calculated separately per treatment arm and over time. For microbiome diversity a diversity trend analysis was performed using Simpson’s diversity index.

### Psoriasis and atopic dermatitis study design (EDP1815-101)

This clinical trial is a first-in-human study of EDP1815 in healthy volunteers, patients with psoriasis, and patients with atopic dermatitis. This randomized, double-blind, placebo-controlled study with dose escalations was designed to assess the safety, tolerability and pharmacodynamic effect of various doses and formulations of EDP1815. The primary objective was safety and tolerability of EDP1815 treatment in each cohort, the secondary objectives were clinical efficacy measures of either psoriasis or atopic dermatitis. As a phase 1 study investigating dose escalations and safety of the investigational medicinal product, the study was not powered for detection of statistical significance of clinical efficacy, but the sample size was selected to determine the initial safety profile of a range of doses of EDP1815, while informing sample size for a subsequent phase 2 study in both psoriasis and atopic dermatitis. Both participants and investigators were blinded to treatment allocation until study completion. 10 cohorts were assessed in this study: cohorts 1–4 and 7 assessed the enteric-coated capsule formulation. Cohorts 5–6 and 8–10 assessed alterations in drug substance or drug product, and therefore results of these cohorts are not presented in this manuscript. Cohorts 1 and 2 were performed in healthy human volunteers, Cohorts 3–4 in patients with mild and moderate psoriasis, and Cohort 7 in patients with mild and moderate atopic dermatitis. The data from these patient cohorts using enteric-coated capsules are presented in this manuscript.

#### Study oversight

This trial was reviewed and approved by the Medicines and Healthcare products Regulatory Agency as a Clinical Trial Application (EudraCT #2018-002807-32) and registered on ClinicalTrials.gov (NCT03733353). The protocol and all patient facing materials including the informed consent form were approved by the Health Research Authority Research Ethics Committee (London-Chelsea). Written and signed informed consent was obtained from all participants prior to their enrollment in the study.

#### Subjects

Main inclusion criteria were healthy participants, other than having the inflammatory skin disease under question in the respective cohort. Subjects were required to be 18 to 65 years of age with a body mass index between 18 and 35 kg/m^2^ and have no known previous exposure to EDP1815. Health status was verified by recording a detailed medical history, a complete physical examination, vital signs, a 12-lead electrocardiogram (ECG) and laboratory testing (including hepatic and renal panels, complete blood count and urinalysis). Participants were excluded in case of any active infection, any GI tract disease that could interfere with drug delivery or GI transit time or having received medications other than paracetamol or antihistamine within 14 days of baseline.

#### Dose selection

The starting dose for the clinical study is based on the predicted therapeutic range based on preclinical *in vitro* and *in vivo* experiments. This expected range is based on the total cell count of microbes given by oral gavage to the mice in the preclinical animal model experiments. This has been adjusted using allometric scaling approaches and converted to a milligram equivalent dose providing an estimate of the likely therapeutic range.

### Statistical analysis of clinical data

Subjects were randomized to EDP1815 or placebo in a 2:1 ratio. Randomization for each cohort was created using a simple block design generated by an unblinded statistician who had no other involvement in the study. The randomization was administered centrally, with the next available randomization number used for each new participant. Investigators and participants were blinded to treatment assignment and all containers and study medication for placebo were identical to those for EDP1815. For Cohorts 3 and 4, a sentinel pair was used to dose one EDP1815 and one placebo participant; safety data for the first 3 days of multiple dosing for the sentinel pair was reviewed prior to the opening of the cohort for further participants. For Cohort 7, the same dose of EDP1815 as was used in Cohort 4 was administered and as such no sentinel dosing was required.

The sample sizes were chosen to explore the tolerability and safety of this new treatment and no formal power calculations were performed. All participants taking at least one dose of study medication were included in the safety analyses. For the efficacy analyses, all randomized participants were included. The protocol did pre-specify that any participants who had an important protocol deviation affecting psoriasis-related efficacy variables would be excluded, but no such deviations occurred.

For Cohorts 3 and 4, where a single dose was administered before being followed up with daily dosing on Day 3, Baseline for efficacy endpoints was assessed as the measurement taken at the Day 3 (start of daily dosing) visit. For Cohort 7, no sentinel dose was used and Baseline was assessed as the measurement taken on Day 1.

Incidences of AEs and SAEs were produced by treatment and severity with separate summaries of study drug-related events. For the continuous efficacy endpoints for skin assessment (LSS and PASI for psoriasis; EASI, IGA*BSA and SCORAD for atopic dermatitis), data was analyzed with a mixed model for repeated measures, including terms for treatment, visit, baseline score and treatment-by-visit interaction. Waterfall plots showing individual percentage changes from baseline were also produced. Patient reported outcomes (DLQI, POEM and pruritis numerical rating scales) were summarized using mean, median, standard deviation, and range. Responder endpoints were summarized using the number and percentage of participants to meet the relevant response definition.

### Psoriasis: study EDP1815-101, cohorts 3 and 4

Two cohorts of 12 and 18 patients with mild to moderate psoriasis, both randomized 2:1 active to matching placebo.

EDP1815 was administered as a single dose (day 1), and after confirming safety, as a once daily for 28 days (days 3–30), with follow-up at day 42. The dose was 1.6 × 10^11^ (cohort 3) or 8.0 × 10^11^ (cohort 4) bacterial cells per day. Placebo subjects were pooled across both cohorts in the analysis.

#### Subjects

The psoriasis-specific inclusion criteria were as follows: patient has a confirmed diagnosis of plaque psoriasis for at least 6 months, and a BSA of 10% or less (excluding the scalp), with at least two psoriatic lesions. Patients were excluded if they had received systemic non-biologic psoriasis therapy within 4 weeks prior to screening, biologic therapy within 12 months prior to screening, or topical agents that could affect psoriasis within 2 weeks of dosing (unmedicated emollient was permitted if the subject was already using this as part of their standard care). Pharmacologically active treatments for psoriasis or atopic dermatitis were not permitted at any timepoint.

#### Safety and tolerability data

The primary endpoint was safety and tolerability. Measurements were adverse events, laboratory assessments (biochemistry including CRP, hematology, urinalysis), physical examination, vital signs, and ECG readings at multiple timepoints, including end of treatment and follow-up. AEs were monitored continuously from screening to follow-up visit.

#### Efficacy data

The secondary endpoints of LSS, BSA, PGA and the PASI score were measured at baseline, weekly until Day 28, and at Day 42.

### Microbiome sequencing

Stool samples were taken at three time points: baseline, day 28, and day 42. Stool was collected in DNA/RNA Shield Fecal Collection tube (Zymo Research) and stored at −80°C until processing. DNA extraction, qPCR and 16S sequencing were performed at and by Baseclear (Leiden, Netherlands) according to their SOPs.

For 16S sequencing, the V4 region of bacterial 16S ribosomal RNA were amplified using universal primer set 515F and 806R (41). Resulting products were sequenced through the Illumina MiSeq platform on a 2 × 250 paired-end run. Reads were demultiplexed and quality filtered before being uploaded to the One Codex platform (42). Paired-end reads were merged and then characterized using One Codex’s in-house Targeted Loci Database, a curated database of bacterial marker genes including 16S ribosomal RNA. Read count and relative abundance tables were calculated at the genus level and retrieved using custom Python scripts and the One Codex Python library.

To determine whether some genera were abundant in Placebo-*vs* EDP1815-treated individuals, read count tables were fed to ANCOM, a statistical framework for the analysis of microbiomes (43). Genera were determined to be significantly different between comparators if mean relative abundance was >1% in either comparator, or they passed the significance threshold identified by ANCOM.

16S sequencing reads were classified at the genera level using the One Codex Platform (42) Reads were grouped by subject ID, treatment and time point using custom Python scripts and the Pandas library (44). Figure was generated using custom python scripts and the python libraries Matplotlib and Seaborn (45, 46).

### Atopic dermatitis: study EDP1815-101 cohort 7

Twenty-four participants with mild and moderate atopic dermatitis were randomized 2:1 active to matching placebo. EDP1815 was administered as 8.0×10^11^ bacterial cells once daily for 56 days, with follow-up at Day 70.

#### Subjects

The atopic dermatitis-specific inclusion criteria were as follows: patient has a confirmed diagnosis of atopic dermatitis for at least 6 months, an IGA score of 2 or 3, and a BSA involvement of 5–40%. Patients were excluded if they had received systemic non-biologic atopic dermatitis therapy within 4 weeks prior to screening, biologic therapy within 12 months prior to screening, or topical agents that could affect atopic dermatitis within 2 weeks of dosing although unmedicated emollient and low potency steroids were permitted if the subject was already using this as part of their standard care.

#### Safety and tolerability data

The primary endpoint was safety and tolerability. Measurements were adverse events, laboratory assessments (biochemistry including CRP, hematology, urinalysis), physical examination, vital signs, and ECG readings at multiple timepoints, including end of treatment and follow-up. AEs were monitored continuously from screening to follow-up visit.

#### Efficacy data

Efficacy was assessed using the clinician reported outcomes of EASI, SCORAD, IGA, BSA, and IGA*BSA, and the patient reported outcomes of DLQI, POEM, and Pruritus-NRS.

## Data availability statement

The data presented in the study are deposited in the NCBI repository, accession number PRJNA899863, https://www.ncbi.nlm.nih.gov/bioproject/PRJNA899863.

## Ethics statement

The studies involving human participants were reviewed and approved by Medical Ethics Committee “Medisch Ethische Toetsingscommissie van de Stichting Beoordeling Ethiek Biomedisch Onderzoek” (Assen, Netherlands) Health Research Authority Research Ethics Committee (London-Chelsea). The patients/participants provided their written informed consent to participate in this study. The animal study was reviewed and approved by Institutional Animal Care and Use Committee (IACUC) at Avastus Preclinical Services (Cambridge, MA).

## Author contributions

AI, KR, TC, MS, MM, DuM, and MB conceptualized and designed studies. AI, KR, DoM, TC, ET, WC, MS, MM, NC, and GM performed experiments, analyzed, and interpreted data. AI, DoM, KR, LW-S, DuM, and MB wrote and edited the manuscript. DoM, MS, MM, and DuM oversaw clinical trial conduct. All authors contributed to the article and approved the submitted version.

## Conflict of interest

AI, DoM, KR, GM, NC, TC, ET, WC, LW-S, SA, DuM, and MB are employees and shareholders of Evelo Biosciences, which sponsored the clinical trials of EDP1815.

The remaining authors declare that the research was conducted in the absence of any commercial or financial relationships that could be construed as a potential conflict of interest.

The authors declare that this study received funding from Evelo Biosciences. The funder had the following involvement in the study: study design, data analysis, decision to publish, and preparation of the manuscript.

## Publisher’s note

All claims expressed in this article are solely those of the authors and do not necessarily represent those of their affiliated organizations, or those of the publisher, the editors and the reviewers. Any product that may be evaluated in this article, or claim that may be made by its manufacturer, is not guaranteed or endorsed by the publisher.
