# Supplementary material for: Clinical translation of anti-inflammatory effects of Prevotella histicola in Th1, Th2, and Th17 inflammation
Source: Front Med (Lausanne). 2023 May 5;10:1070433. doi: 10.3389/fmed.2023.1070433 (PMC10197930; doi:10.3389/fmed.2023.1070433)

**Supplementary Methods**

**Mouse biodistributi****on studies**

***Dosage and Administration of Bacteria.*** Bacteria were grown in tryptic soy broth supplemented with hemin and vitamin K and incubated overnight for 16-24 hours in an anaerobic chamber (5% H_2_, 5% CO_2_, 90% N_2_) at 37°C. Bacteria were suspended in anaerobic PBS to a concentration appropriate for dosage and used within 30 min. C57BL/6 mice were dosed orally with 200ul anaerobic PBS or 200ul of a 1x10^10^ cells/ml suspension of EDP1815. At 1, 4, 8-, 16-, 24-, and 32-hours post-gavage, a stool sample, 5 cm of ileum, and 3 cm of colon were collected from each mouse dosed with EDP1815. Samples from mice dosed with anaerobic PBS were collected only at 4 hours post-gavage. Tissues were stored at -80°C until DNA extraction.

***DNA Isolation and qPCR.*** Bacterial DNA was isolated from the *in vitro* culture using the GenElute Bacterial Genomic DNA Kit (Sigma Aldrich). DNA was isolated from tissue from the QIAamp PowerFecal DNA kit. Total number of bacteria in each sample was determined with qPCR using EDP1815 strain-specific and universal bacterial 16S primers. All qPCR reactions were performed using 2X SsoAdvanced Universal Probes Supermix and contained 10 µM of both the forward and reverse primers and 5 µM probe. Standards for the qPCR reactions were prepared using a known amount of gBlock serially diluted in PCR grade water. To account for differences in total DNA isolated, the number of EDP1815 and total bacteria (16S) in the sample obtained by qPCR were normalized to the total nanograms of DNA obtained from each tissue.

***Custom qPCR Reagents Used in qPCR.***

ES894 Forward Primer-5’-TTGGCTTCCTGTACTCCTCG-3’, ES894 Reverse Primer**- 5’-**TCCGGACAAATCTGTGACGA-3’, ES894 probe labelled with Fam on the 5’ End-5’- /56-FAM/TCACCCTAATTCGCTGATCCTTCTTCGCA-3’, ES894 gBlock fragment

for Standard Curve- 5’-CAGCCCCCCTTGGCTTCCTGTACTCCTCGTCTTCGTGTAGC

ATTTATTCCTTCATTATTTTTATGTATCACCCTAATTCGCTGATCCTTCTTCGCATATTCGTCACAGATTTGTCCGGACTTATCTAAACT-3’. The BacQuant 16S primers were obtained from this previously reported study(*25*)

**Human Biodistribution studies**

Samples were also taken to check for systemic presence of EDP1815 (blood qPCR), and microbiome analysis (stool sample for qPCR and 16SsPCR).

***Blood qPCR***

DNA was extracted using the PAXgene Blood DNA Kit (Qiagen, Cat#761133) following the manufacturer’s instructions. The resulting DNA extracts were normalized to 5ng total DNA per qPCR reaction.

Primer and probe sequences specific to EDP-1815 are listed below:

EDP-1815 Forward Primer: 5’-TTGGCTTCCTGTACTCCTCG-3'

EDP-1815 Reverse Primer: 5’-TCCGGACAAATCTGTGACGA-3’

EDP-1815 probe: 5’-/56-FAM/TCACCCTAATTCGCTGATCCTTCTTCGCA-3’

For each PCR reaction, PerfeCTa® qPCR Tough Mix®, Low ROX™ (QuantaBio, Cat#95114-012), 500 nM of each primer set, 250 nM of probe and 5ng of DNA in 10uL were denatured at 95oC for 10 minutes, followed by 40 cycles of denaturation at 95oC for 15 seconds and annealing and elongation at 60oC for 60 seconds. All experiments were performed using Applied Biosystems MicroAmp Fast Optical Reaction plates, sealed with MicroAmp Optical adhesive films. All samples were run in triplicate. These experiments were run using an Applied Biosystems QuantStudio7 Flex and ProSystem Real-Time PCR machine. Analysis was performed using Applied Biosystems QuantStudio Real-Time PCR Software v1.3.

***Stool qPCR***

DNA extraction was performed using the Quick-DNA™ Fecal/Soil Microbe Miniprep Kit (Zymo Research) according to manufacturer’s instructions using bead beating for mechanical lysis with 1000μl fecal slurry.

Extracted DNAs were diluted to 0.8 ng/μl in deep well plates (Kisker) and sealed using a MicroAmp Clear Adhesive Film (Thermo Fisher Scientific). All qPCR reactions were performed in 384 well PCR plates (Thermo Fisher Scientific) sealed with MicroAmpOptical Adhesive Film (Thermo Fisher Scientific) using anApplied Biosystems QuantStudioTM5 Real-Time PCR system (Thermo Fisher Scientific) with QuantStudioTMDesign & Analysis software v1.4.2.

Each qPCR reaction was carried out in a total volume of 10μl, using 5 μl SsoAdvancedTM, 0.5 μl EDP1815 Forward primer (10 μM), 0.5 μl EDP1815 Reverse primer (10 μM), EDP1815 probe (5 μM) and 2ng diluted template DNA. The primers and probe sequences are the same as shown in the previous “Blood qPCR” section.

The PCR program started with a denaturation step at 95°C for 10 minutes, followed by 40 cycles consisting of denaturation at 95°C for 15 s, annealing and elongation at 58°C for 1 minute.

**Supplementary Figure 1 Legend**

Biodistribution in mice. Copies of DNA per ng of total DNA at each time point after a single orally administered dose (EDP1815; TCC-4.69E+09), in the ileum, colon and stool. The blue line represents DNA for EDP1815, the dotted black line represents total commensal bacterial load as measured by 16S sequencing, and the grey line represents the lower level of detection for the assay. n = 3 at each timepoint. LOD of 6x10^-3^ copies/ng DNA.

**Supplementary Figure 2 Legend**

Stacked bar plot of ten most abundant genera across all timepoints (baseline, last dose and washout) for subjects dosed with EDP1815 or placebo.


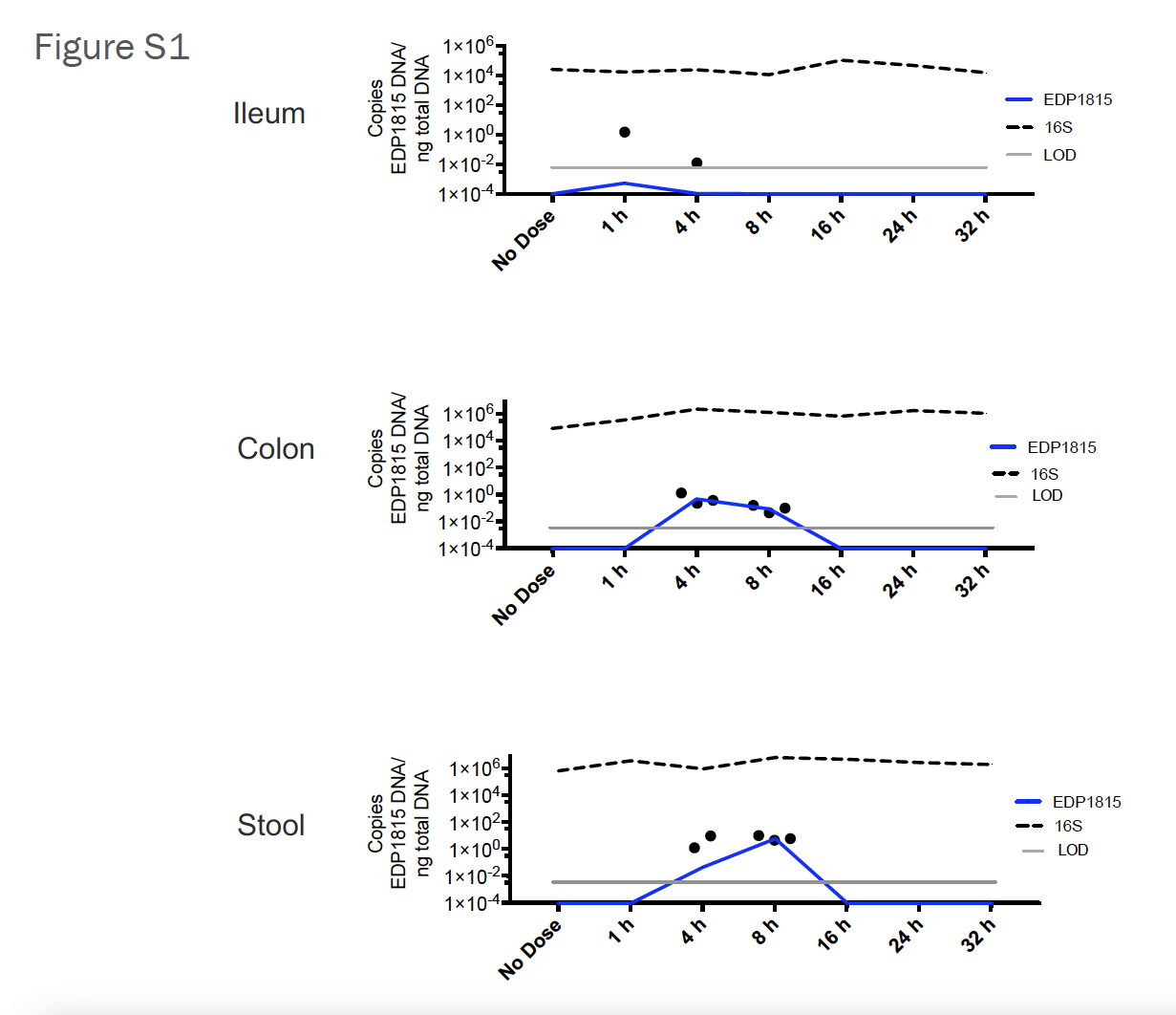


Figure S2


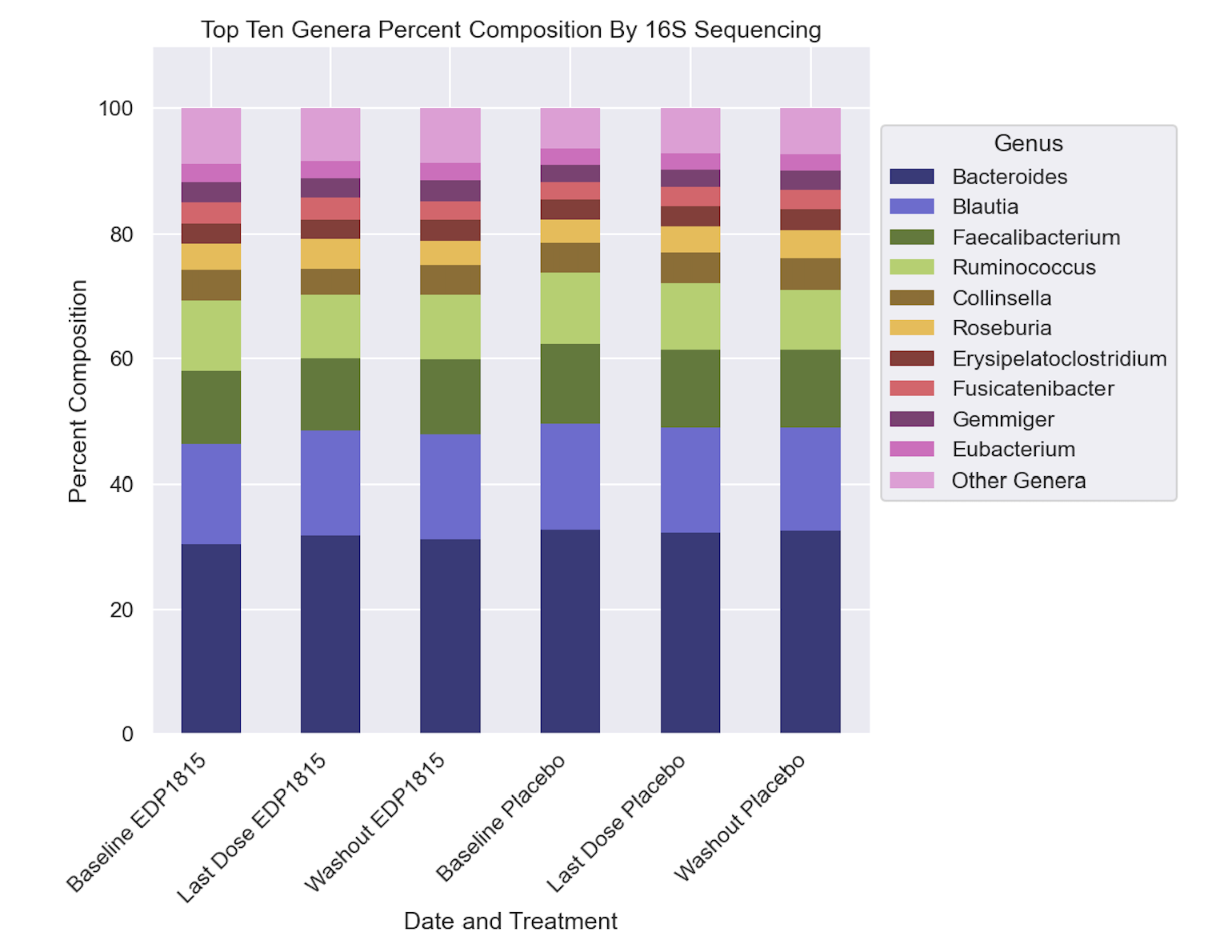

Supplement: Supplementary file 1 [file Data_Sheet_1.docx]
